# Efficacy of Asymmetric Myopic Peripheral Defocus Lenses in Spanish Children: 24-Month Randomized Clinical Trial Results

**DOI:** 10.3390/children12020191

**Published:** 2025-02-06

**Authors:** Clara Martinez-Perez, Miguel Ángel Sánchez-Tena, Jose Miguel Cleva, Cesar Villa-Collar, Marta Álvarez, Eva Chamorro, Cristina Alvarez-Peregrina

**Affiliations:** 1 School of Management, Engineering and Aeronautics, ISEC LISBOA—Instituto Superior de Educação e Ciências, Alameda das Linhas de Torres, 179, 1750-142 Lisbon, Portugal; masancheztena@ucm.es; 2 Department of Optometry and Vision, Faculty of Optics and Optometry, Universidad Complutense de Madrid, 28037 Madrid, Spain; cristina_alvarez@ucm.es; 3 Clinical Research Department, Indizen Optical Technologies, 28002 Madrid, Spain; jmcleva@iot.es (J.M.C.); malvarez@iot.es (M.Á.); evachamorro@iot.es (E.C.); 4 Faculty of Biomedical and Health Science, Universidad Europea de Madrid, 28670 Madrid, Spain; villacollarc@gmail.com

**Keywords:** myopia, axial length, spectacle lenses

## Abstract

**Background/Objectives**: Asymmetric myopic peripheral defocus lenses (MPDLs) have proven to be effective in slowing the progression of myopia in Spanish children over a period of 12 months. The purpose of this study was to assess the MPDL spectacles’ efficacy in slowing myopia progression over a 24-month period in children. **Methods**: This study extends the follow-up period of the double-masked, prospective, and randomized clinical trial previously published to 24 months. Children from 6 to 12 years were assigned to two groups: a control group wearing spherotorical single vision lenses (SVLs) or a treatment group wearing MPDL lenses. Inclusion criteria included children with myopia less than −0.50 D, astigmatism below 1.50 D, and best-corrected visual acuity of at least 20/20. Participants underwent cycloplegic autorefractive examination and axial length (AL) measurements at the baseline and six and twelve months in the study already published, and twenty-four months later in the present study. Lifestyle factors, including outdoor activities and digital device use, were also assessed. Baseline characteristics, including age, refractive error, and AL, were comparable between groups. Dropout rates were 15.9%, with 14 participants lost to follow-up, distributed equally between the two groups. **Results**: After 24 months of follow-up, 69 children remained in this study, comprising 34 participants in the SVL cohort and 35 in the MPDL cohort. Over 24 months, the MPDL group showed significantly less AL elongation than the SVL group (0.27 ± 0.23 mm and 0.37 ± 0.24 mm; *p* = 0.0341). The mean relative AL increase was 1.10 ± 0.95% in the MPDL group, compared to 1.56 ± 1.02% in the SVL group (*p* = 0.0322). Younger children exhibited faster AL growth, while digital device use and outdoor activities did not affect AL changes. **Conclusions**: MPDL spectacle lenses substantially slowed myopia progression over a 24-month period, with 28.7% less progression in absolute AL growth and 29.8% in relative AL growth compared to SVL. These results indicate that MPDL lenses are an effective method for slowing myopia progression.

## 1. Introduction

One of the most prevalent ocular disorders globally is myopia [1,2,3], and its incidence rates are rapidly increasing across the globe [4,5]. The earlier onset of myopia is strongly associated with the progression to severe levels of myopia in adulthood [5,6]. Low and moderate myopia can develop into high myopia of −6.00 D or greater if not addressed in time [7]. This progression can result in severe long-term ocular health issues. Severe myopia has been linked to an increased likelihood of experiencing complications such as glaucoma, myopic macular degeneration, retinal detachment and, in extreme cases, irreversible vision loss [8].

The global incidence of high myopia is expected to rise considerably, with an increase estimated at 117 million people from 2020 to 2030 [3,9]. As a result, pathological myopia is projected to become the main cause of blindness and irreversible vision impairment in the world. The growing concern regarding the potential complications associated with high myopia has intensified the call for effective myopia control measures. Early detection and effective intervention to manage myopia and slow its progression, particularly in children, have thus become critical.

The initiation and progression of myopia are governed by a combination of visual, environmental, and genetic factors that modulate eye growth in response to visual inputs [10]. Research involving animal models has been instrumental in deepening our knowledge of myopia and advancing the development of effective treatment approaches [11]. Research using these models has demonstrated that emmetropization is a visually guided process, where myopic defocus decelerates eye growth and hyperopic defocus accelerates it [12]. Research on animals such as rhesus monkeys [13], marmosets [14], chicks [15], and guinea pigs [16] has shown that myopic defocus, induced by multifocal or dual-power lenses, can slow down or even reverse eye growth associated with myopia. This principle forms the basis of various myopia control strategies, including orthokeratology (OK), spectacle lenses, and multifocal soft contact lenses.

A recent meta-analysis reviewed the effectiveness of current treatments, both pharmacological and optical alternatives, revealing their efficacy in slowing myopia progression by reducing refractive changes and axial length (AL) elongation [17]. Optical interventions for myopia management such as spectacle lenses, soft contact lenses, and OK work by modifying retinal defocus, specifically inducing myopic defocus to slow the progression of myopia. The meta-analysis concluded that these optical interventions are effective in managing childhood myopia, with the most significant effects observed in the first 12 months of use, followed by a decrease in effectiveness over time [18].

Recently developed ophthalmic spectacle lenses designed to control myopia progression can be grouped into three categories: simultaneous defocus, contrast management, and peripheral addition designs [18,19]. The first group includes lenses like defocus incorporated multiple segment (DIMS) [20], highly aspherical lenslets target (HALT) [21,22], and cylindrical annular refractive element (CARE) [23] lenses. The second group is composed of the diffusion optics technology (DOT) lenses [24]. The third group includes lenses that produce progressive peripheral defocus [25,26,27,28,29].

In a previously published study [29], the effectiveness of a new ophthalmic lens design featuring asymmetric myopic peripheral defocus (MPDL, MyoLess^®^, IOT, Madrid, Spain) was evaluated over 12 months in a cohort of Spanish children. The results showed a significant reduction in AL elongation for the group treated with MPDL compared to the control group with single-vision lenses (SVLs). After one year, the MPDL group exhibited an AL elongation of 0.14 ± 0.14 mm, compared to 0.23 ± 0.15 mm in the control group, representing a 39% reduction in absolute AL growth (*p* = 0.014).

The current research builds on previous work, aiming to expand it to a two-year period to examine the sustained efficacy of MPDL lenses in slowing the progression of myopia.

## 2. Materials and Methods

This is a double-masked, prospective, randomized clinical trial conducted at Novovision (Madrid, Spain). The study was initially planned to follow children for one year to evaluate the efficacy of MDPL lenses for myopia control. This is an extension of the follow-up to 24 months, following the same methodology already published [29].

The inclusion criteria were children aged five to twelve years with a cycloplegic spherical equivalent refraction (SE) less than or equal to 0.50 diopters, astigmatism less than or equal to 1.50 diopters, anisometropia less than or equal to 1.50 diopters, and corrected visual acuity (VA) greater than or equal to 20/20.

Children were randomly assigned at the beginning of the study to the intervention group fitted with MPDL lenses and the control group using SVL. The MPDL lenses have a 7 mm central zone free of defocus and an asymmetric myopic defocus zone of +1.50 D at 25 mm nasally, +1.80 D at 25 mm temporally, and +2.00 D in the lower region. The lenses were made using freeform technology and were customized for each child based on their prescription, pupillary distances, and pupillary heights. The defocus pattern was identical for all children.

The Ethics Committee of Hospital Clínico San Carlos in Madrid, Spain, granted approval for the randomized controlled trial, ensuring compliance with the ethical guidelines outlined in the Declaration of Helsinki. Additionally, the trial was officially documented on ClinicalTrials.gov under the registration ID NCT05250206.

### 2.1. Visual Examination

At the 24-month visit, SE, AL, and VA evaluations were performed following identical criteria to previous follow-up visits. For SE, the Canon Full Auto Ref-keratometer RK-F1 (Canon, Tokyo, Japan) was used after administering three drops of cyclopentolate to induce cycloplegia. For AL, the IOL Master^®^ optical biometer (Carl Zeiss Meditec, Jena, Germany) was used. The best corrected VA was measured monocularly at distance vision. Additionally, data from the baseline visit were considered for determining factors associated with AL growth: demographics (age and gender), family history of myopia (number of parents with myopia), and children’s lifestyle (time on digital devices and time outdoors). Table 1 shows the data collected at each visit.

### 2.2. Statistical Analysis

The relative (%) and absolute (mm) AL values were statistically compared between children wearing MPDL and SVL lenses. Relative AL growth refers to the percentage change in AL relative to the baseline measurement for each individual. It was calculated using the following formula:

(AL at 24 months−baseline AL)baseline AL

Statistical analysis was conducted using Python (version 3.13.1). Continuous variables were analyzed according to their distribution: means were analyzed through Student’s *t*-test, while median values were analyzed employing the Mann–Whitney U statistical method. Categorical data, including factors like gender distribution and parental myopia, were assessed using the Chi-square test. To identify factors associated with relative AL growth, such as age and other sociodemographic and lifestyle factors, univariate and multivariate linear regression analyses were performed. The significance level was set at a *p*-value of less than 0.05.

## 3. Results

**Figure 1:**
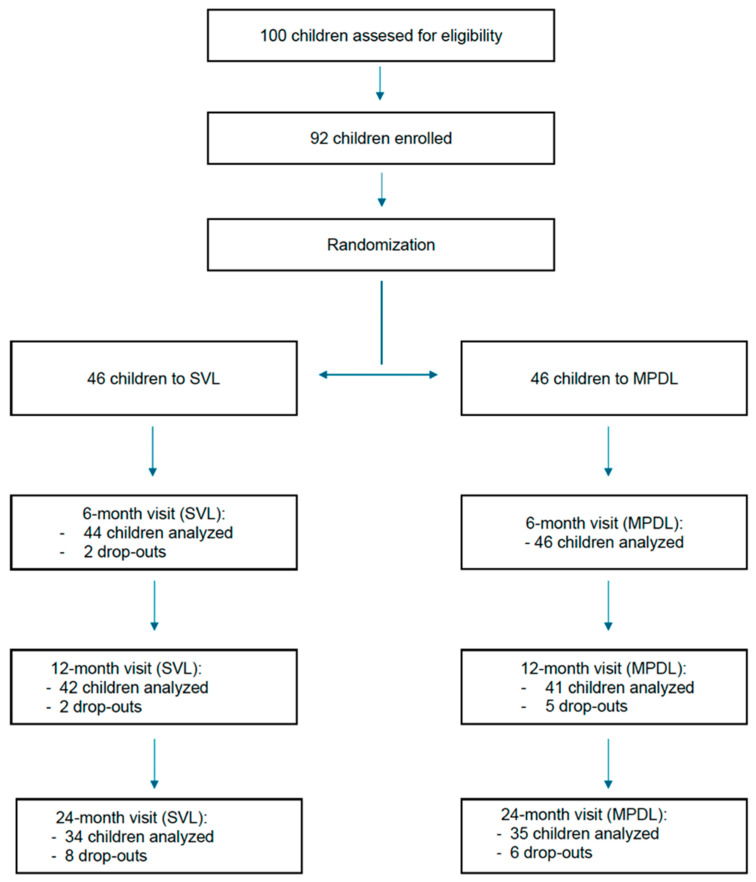
Revised flowchart of the randomized clinical trial illustrating participant allocation and follow-up visits over the 24-month period. MPDL: asymmetric peripheral myopic defocus lens; SVL: single-vision lens.

The baseline and one-year results of this study have already been published by Sánchez-Tena et al. [29]. Initially, 92 children were recruited and randomly assigned to two groups: the SVL and MPDL groups were the control and treatment, respectively. During the first year, nine participants dropped out (five from the MPDL cohort and four from the SVL cohort), leading to an attrition rate of approximately 10%. The analysis included 83 children (42 with SVL and 41 with MPDL). In the second year, an additional 14 children left the study (6 from the MPDL cohort and 8 from the SVL cohort). Factors contributing to withdrawal included starting a new treatment after the 12-month study (six children from each group), relocating (one child from the control group), and missing follow-up visits (one child from the control group) (Table 2). Ultimately, 69 children completed the two-year follow-up (35 with MPDL and 34 with SVL), with a mean age of 10.0 ± 1.8 years (ranging from 6 to 12 years). Power analysis determined that a sample size of 34 participants per group would provide 80% power, based on a one-sided test with a 5% Type I error threshold. This calculation assumes a mean difference between groups of 0.14 mm with a standard deviation (SD) of 0.23 mm. The flowchart of the RCT is shown in Figure 1, and the baseline demographic and clinical characteristics of the participants are presented in Table 3.

**Figure 2:**
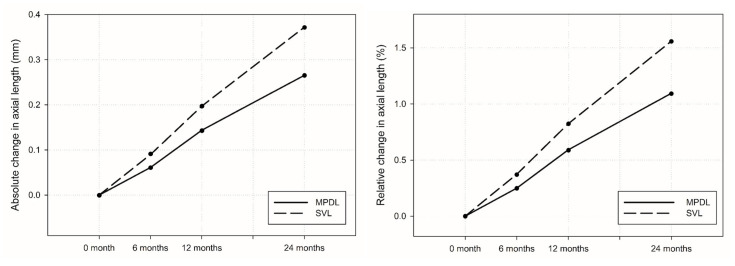
Absolute and relative axial length change from the baseline to 24 months; SVL: single-vision lens; MPDL: asymmetric myopic peripheral defocus lens.

After two years of follow-up, no significant differences were observed between the two eyes; therefore, only data from the right eye were used for statistical analysis (Table 4 and Figure 2). Over the 24-month period, the SVL group exhibited an AL increase of 0.37 ± 0.24 mm (95% CI: 0.29 to 0.45 mm), whereas the MPDL group showed a smaller increase of 0.27 ± 0.23 mm (95% CI: 0.19 to 0.35 mm; *p* = 0.0341). The absolute difference in AL elongation between the groups was 0.10 mm (95% CI: −0.01 to 0.21 mm). This corresponded to a median relative growth of 1.32% [0.93–2.12] in the SVL group versus 0.93% [0.42–1.72] in the MPDL group (*p* = 0.0322). The mean relative AL increase was 1.10 ± 0.95% (95% CI: 0.77 to 1.43%) in the MPDL group, while it was 1.56 ± 1.02% (95% CI: 1.20 to 1.92%) in the SVL group. The use of MPDL lenses resulted in a significant reduction in absolute AL growth by 28.7% and in relative mean AL growth by 29.8% compared to the SVL group.

The change in mean cycloplegic refraction at the 2-year follow-up was 0.43 ± 0.49 D in the control group (95% CI: 0.26 to 0.60 D) and 0.20 ± 0.43 D in the MPDL group (95% CI: 0.05 to 0.35 D).

**Figure 3:**
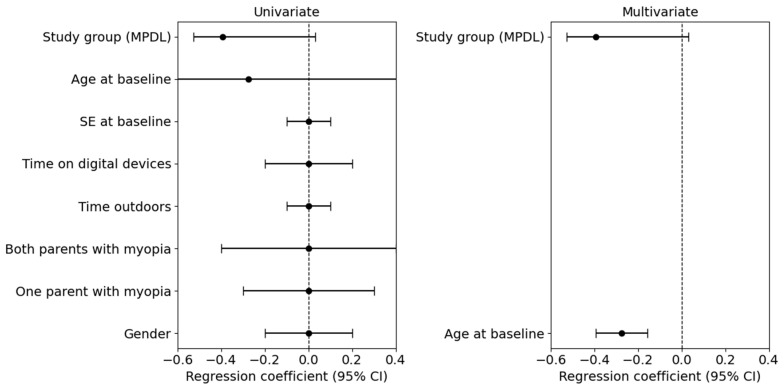
Factors related to AL relative growth at 24 months.

Regarding the factors linked to relative AL growth, univariate linear regression analyses revealed that associations with variables such as gender, parental myopia, outdoor time, hours spent using digital devices, and the initial spherical equivalent had no significant impact on the relative AL growth between the baseline and 24 months. However, the age of the child at enrollment affected the proportional increase in axial length during both periods (regression coefficient for baseline to 24 months: −0.275, 95% CI: −2.232 to 1.681). The negative regression coefficient (−0.275) indicates that for each additional year of age at the start of the study, the relative AL growth was lower. This suggests that AL increases more in younger children, which could imply that myopia progression is faster in this group. As such, the efficacy of the MPDL lens in controlling AL growth may be greater in younger children, who experience a faster progression rate. The regression coefficient was −0.275, with a 95% CI of −0.393 to −0.158 for the period between the baseline and 24 months (Figure 3).

## 4. Discussion

In this study, it was found that after two years, MPDL spectacle lenses reduced the absolute AL growth by 28.7% and the relative AL growth by 29.8% compared to SVL, suggesting that MPDL lenses maintain efficacy over time and provide sustained intervention to slow myopia progression. The current study provides results after 24 months of treatment from the first RCT carried out in a Caucasian population, evaluating MPDL lenses. Note that when comparing our results with other studies on the efficacy of myopia control lenses over two years, we observed several differences that may be attributed to population characteristics. Currently, to our knowledge, there are few published studies assessing the two-year efficacy of myopia control spectacle lenses in European populations.

Lam et al. [20] carried out research on the efficacy of lenses incorporating defocus incorporated multiple segments (DIMSs) within a population of Chinese participants. These lenses are designed with a central 9 mm aperture, surrounded by a 33 mm treatment zone embedded with multiple +3.5 D positive power lenslets. The results after two years showed a change of 0.55 ± 0.02 mm in AL for the SV group, compared to 0.21 ± 0.02 mm for the treatment group, resulting in a difference of 0.34 mm.

Similarly, Bao et al. [22] investigated the effectiveness of HALT lenses in a population of Chinese participants. These lenses are designed with a central zone dedicated to full prescription correction and a 10 mm diameter aperture at the core. This central area is encircled by a treatment zone comprising 11 concentric rings, each containing aspherical lenslets measuring 1.1 mm in diameter. These lenslets have positive powers ranging from +3.50 D in the peripheral rings to +6.00 D in the central rings. After 2 years of follow-up, they found an AL increase of 0.69 ± 0.04 mm in the SV group, compared to 0.34 ± 0.03 mm in the treatment group, with a difference of 0.35 mm.

In contrast, studies conducted in non-Asian populations have shown somewhat different results. The study by Rappon et al. [30] on DOT lenses in the United States observed that after two years of follow-up, there was an AL increase of 0.53 ± 0.33 mm in the SV group, compared to 0.33 ± 0.23 mm in the treatment group, with a difference of 0.20 mm. DOT technology features a central zone with a diameter of 5 mm, surrounded by translucent microscopic diffusers (0.14 mm in diameter) designed to scatter light. This helps reduce contrast and, consequently, reduces the disparity in activity between the L and M cones.

A recent study by Lembo et al. [31] evaluated the effectiveness of DIMS and HALT lenses in slowing myopia progression over a 2-year period in a European population. This retrospective cohort study assessed axial length (AL) elongation in two groups: 73 children wearing DIMS lenses (mean age: 11.2 ± 2.3 years) and 73 children wearing HALT lenses (mean age: 11.4 ± 2.4 years). At the 2-year follow-up, the mean AL increase was 0.29 ± 0.63 mm for the DIMS group and 0.32 ± 0.72 mm for the HALT group. Similar findings have been reported in other studies on European populations evaluating myopia control interventions using contact lenses. For instance, the study by Ruiz-Pomeda et al. [32] analyzed the efficacy of MiSight lenses in a Spanish cohort of 46 children (mean age: 11.0 ± 1.2 years) and observed a mean AL increase of 0.28 mm (95% CI: 0.20 to 0.37) over 2 years in the MiSight group. Additionally, the multicenter study by Chamberlain et al. [33] reported that a group of 70 children (mean age: 10.1 ± 1.3 years) treated with MiSight contact lenses exhibited a mean AL increase of 0.23 ± 0.03 mm over 2 years. These results align with our findings in children wearing MPDL lenses (mean age: 10.4 ± 1.8 years), where the mean AL elongation was 0.27 ± 0.23 mm over the same period.

Our study showed a variation after two years of follow-up of 0.27 ± 0.23 mm in AL in the MPDL group and 0.37 ± 0.24 mm in the SVL group, which resulted in a difference of 0.10 mm or an efficacy of 29% in reducing AL growth, which are somewhat lower than the effects observed in the Asian studies. Lam et al. [34] reported a 62% reduction in AL elongation with DIMS lenses over a two-year period, while Gao et al. [35] showed that HALT lenses achieved a reduction of approximately 51%. These differences could be attributed to the specific population characteristics in our study. In this sense, it is well-established that the progression of myopia varies significantly between Asiatic and non-Asiatic populations. Studies have consistently shown that individuals of East Asian descent, particularly those from countries such as China, Japan, and South Korea, tend to experience more rapid myopia progression compared to populations from Western countries [36,37]. This difference in progression rates has been attributed to a combination of genetic, environmental, and lifestyle factors. In East Asia, the high prevalence of myopia is often linked to increased near-work activities, such as additional time spent on reading, studying, and using digital devices, as well as limited outdoor activity [38,39]. In contrast, populations from non-Asian regions, such as Europe and North America, generally show slower rates of myopia progression [40]. In Spain, for instance, the greater number of sunlight hours and a lifestyle that often involves more outdoor activities may contribute to a slower progression of myopia. Children in Spain are typically exposed to more natural light, which has been suggested to have a protective effect against myopia progression [41,42]. These differences in progression rates highlight the need to consider ethnicity, lifestyle, and environmental factors when assessing the efficacy of myopia control interventions.

On the other hand, our cohort represents a population with a relatively lower risk of myopia progression compared to what is typically reported in the literature. In our study, children in the control group experienced an increase of 0.37 mm in AL after two years of follow-up. However, according to a model for non-Asian populations [43], an increase of 0.46 mm in AL would be expected over the same period in a population with an average age, like that of our sample. This aligns with the characteristics of a lower-risk population, where the progression of myopia tends to be slower.

Additionally, it is important to consider potential biases introduced by dropouts in the study. Data from children who completed the one-year follow-up [29] showed an increase in AL of 0.14 mm in the MPDL group and 0.23 mm in the control group. The control group values were consistent with the virtual control group, which predicts an AL growth of 0.23 mm after one year of follow-up for children with an average age equivalent to that of our sample. However, in the remaining sample that completed the two-year follow-up, the AL growth of the control group was considerably smaller than expected in the virtual control group. Specifically, our study showed an increase in AL of 0.14 mm in the MPDL group and 0.20 mm in the control group after 1 year of follow-up and, according to the model, an increase of 0.25 mm in AL for the control group for the same period would be expected. Therefore, while the 0.1 mm reduction in AL elongation observed in our study is modest compared to other interventions, it is consistent with the effects expected in a low-risk population.

When comparing optical treatments with pharmacological ones, it is evident that both have advantages and limitations in controlling myopia progression. MPDL demonstrated a significant 29–30% reduction in myopia progression in our study. Pharmacological therapies such as low-concentration atropine (0.05%) have shown greater efficacy in several studies, achieving up to a 50% reduction in myopia progression over two years [44,45]. However, it is important to note that many of these studies, such as the LAMP2 trial, were conducted in Asian populations, where the effects of atropine may differ compared to non-Asian populations [44]. On the other hand, although atropine could be more effective, this therapy presents drawbacks such as the need for daily instillation, side effects like photophobia and near-focusing difficulties, and potential adherence barriers in children and families. In contrast, MPDL lenses offer a non-invasive option with better acceptance among parents and children, as they eliminate the need for topical medications. The use of spectacle lenses for myopia management has been extensively studied in recent years and is currently one of the preferred options for myopia control due to their ease of fitting, even for younger children. Additionally, spectacles generally have fewer or no side effects compared to other optical treatments like contact lenses or atropine. This balance between convenience, side effects, and efficacy must be carefully considered when choosing a treatment, as each approach can be tailored to the individual needs and preferences of patients and their families. Future studies could further explore parents’ perceptions of these treatments and how these preferences influence therapy selection.

Regarding lens design, most of the lenses discussed feature a central zone that fully corrects the prescription, with the diameter varying by design, and a peripheral zone with a positive addition to create myopic blur on the peripheral retina. These spectacle lenses are designed to induce simultaneous myopic retinal defocus for both distance and near vision. The central optical zone typically ranges from 7 to 10 mm in diameter, surrounded by an addition of 1.50 D to 3.80 D. A smaller central zone diameter can cause visual discomfort and issues when wearing the lenses. Another crucial aspect of lens design is the myopic defocus, as its magnitude and distribution can significantly impact lens performance. Previous research has indicated that varying peripheral retinal defocus is vital in controlling myopia progression [46,47]. Additionally, studies have shown that high-add power multifocal contact lenses significantly reduce the rate of myopia progression compared to medium-add power lenses [48]. MPDL is a freeform lens with an optical zone of 7 mm and an asymmetrical progressive peripheral positive defocus of +1.50 D at 25 mm nasally, +1.80 D at 25 mm temporally, and +2.00 D in the lower region. The lenses were designed with the goal of finding an optimal configuration that minimizes the optical area while maximizing the region with plus power and reaching a high level of defocus power that is wearable for the user in order to ensure compliance with the treatment. The positive power added to the lower part of the lens serves to expand the plus defocus area for distance vision while maintaining appropriate comfort for near vision. The asymmetrical positive defocus between the nasal and temporal areas of the lens was incorporated to better align with the morphological characteristics of the myopic retina. It is well-established that the retina exhibits asymmetry, particularly between the nasal and temporal retinal hemifields, with differences observed in anatomical neural features, peripheral refraction, and AL growth [49,50,51]. It has been proposed that retinal sensitivity may differ between the nasal and temporal hemifields, with the nasal region potentially playing a more crucial role in the mechanisms driving eye growth in response to hyperopic defocus [51]. To determine the final power distribution of the lens, various prototypes were developed and tested in a series of trials with young adults. The lens selected for this study was the one that provided the best balance between comfort and design characteristics.

Finally, several factors of the present study have been identified as limitations and deserve discussion. Firstly, baseline data show a significant difference in the mean age between the two groups, with children in the MPDL group being slightly older than those in the SVL group. Although this age difference persisted throughout the 24-month period, our analyses controlled for this variable to ensure that the observed differences in myopia progression were not solely influenced by age. Secondly, while the study duration of two years is considerable, it is shorter compared to other studies, such as the CYPRESS trial evaluating DOT lenses over four years [52], or DIMS studies reporting data spanning up to six years in Chinese pediatric populations [53]. A longer evaluation period could provide a more comprehensive perspective on the long-term efficacy and safety of MPDL lenses in controlling myopia. Another limitation is the dropout rate of 16.7%, which could affect the robustness of the results. Initially, this study was planned for one year, but the extension to two years led to some participants discontinuing treatment after the first year. Furthermore, this study was conducted at a single center in Madrid, which may limit the generalizability of the findings to other regions with different demographic and environmental characteristics. However, the inclusion of a diverse sample in terms of age and gender improves the generalizability of the findings despite these limitations.

## 5. Conclusions

This research is the first two-year, randomized, double-blind clinical trial in Europe to evaluate the use of ophthalmic lenses with an asymmetric myopic peripheral defocus design. The results confirm the effectiveness of MPDL spectacle lenses in controlling myopia in a cohort of Spanish children, demonstrating a 28.7% reduction in the absolute growth in AL and a 29.8% reduction in relative AL growth compared to the control group wearing SVL. These results are clinically relevant, as they provide optometrists with a non-invasive, spectacle-based option for managing myopia progression in children. Given their efficacy and ease of use, MPDL lenses could serve as an alternative or complement to pharmacological interventions, particularly for children or families preferring non-pharmacological approaches.

Future research should focus on validating these findings through multi-center trials that encompass diverse populations and environmental conditions. Longer follow-up periods would also help to assess the sustained efficacy and safety of MPDL lenses, as well as their long-term impact on the risk of developing high myopia and associated complications.

## Figures and Tables

**Table 1:** Assessment and examination schedule.

Visit		Baseline	Dispensing	6 Months	12 Months	24 Months
Signed parental consent form		X				
Basic information	Demographic data	X				
	Medical records	X				
Refraction	Subjective refraction	X		X	X	X
	Cycloplegic autorefraction	X		X	X	X
VA	Uncorrected VA	X		X	X	
	Best-corrected VA	X		X	X	X
Eye evaluation	Keratometry	X				
	Biomicroscopy	X				
	Ocular fundus	X				
	IOP	X				
	AL	X		X	X	X
Binocular vision	Accommodation lag evaluation	X		X	X	
	Accommodation amplitude			X	X	
	Distance and near phorias	X		X	X	
Spectacle fitting	Evaluation of frame alignment and lens condition		X			
Questionnaires	Lifestyle	X				
	Wearability			X	X	

VA: visual acuity; IOP: Intraocular pressure; AL: axial length.

**Table 2:** Summary of dropout reasons and potential impacts on statistical power.

Reason for Dropout	SVL Group (n)	MPDL Group (n)	Total (n)	Potential Impact on Statistical Power
Started a new treatment after 12 months	6	6	12	May introduce bias if participants had faster progression rates.
Relocated	1	0	1	Minimal impact due to low frequency.
Missed follow-up visits	1	0	1	Minimal impact due to low frequency.
Total	8	6	14	Attrition rate remains below threshold to affect power significantly.

**Table 3:** Baseline characteristics of the sample.

	MPDL	SVL	*p*-Value
Mean age (years)	10.4 ± 1.8	9.6 ± 1.8	0.046 *
Median age (years)	11.0 [2.5]	10.0 [2.0]	
Age range (years)	6–12	6–12	
Gender distribution			
Female	23	17	0.274 ^‡^
Male	12	17	
Mean axial length (mm)	24.12 ± 0.94	23.94 ± 0.84	0.400 ^†^
Median axial length (mm)	24.05 [1.44]	23.83 [1.23]	
Mean cycloplegic refraction (D)	−2.57 ± 1.21	−2.00 ± 1.04	0.039 ^†^
Median cycloplegic refraction (D)	−2.50 [1.63]	−1.81 [1.72]	
Family history of myopia			
One myopic parent	15	13	0.187 ^‡^
Both myopic parents	10	16	
Neither parent	9	5	

* U—Mann–Whitney; ^†^ *t*—Student; ^‡^ Chi—cuadrado.

**Table 4:** Axial length change at 2 years: Comparison between SVL and MPDL.

	SVL	MPDL	*p*-Value
Change in Axial Length between Initial and 24 Months			
Absolute change (mean ± SD) (mm)	0.37 ± 0.24	0.27 ± 0.23	0.0341
Relative change (mean ± SD) (%)	1.56 ± 1.02	1.10 ± 0.95	0.0322
Relative change (median [Q1, Q3]) (%)	1.32 [0.93–2.12]	0.93 [0.42–1.72]	0.0322

## Data Availability

The data generated in this study can be requested from the corresponding author. However, public access is restricted due to ethical considerations and the need to maintain participant confidentiality.
